# Bashful Bridegrooms

**Published:** 1892-06

**Authors:** 


### BASHFUL BRIDEGROOMS.

I was once (says a writer in the *Philadelphia Call*) "best man" to a stalwart, middle-aged bridegroom, noted for his courage and feats of daring, and when the time came for us to go down stairs to meet the bride and her attendants, he nearly had a fit, and he looked like a walking corpse all through the ceremony. I had to keep saying: "Brace up, old boy!" and "Come, come, you've got to go down!" to get him started, and at the door he was idiotic enough to clutch at me and say: "Say, Fred, how would it do to have Mary and the preacher slip in here and have it all over with before we go down at all? I can't go through it before all that crowd." "Idiot," I said, pointedly enough to leave no doubt as to my meaning, "Mary won't come in here, and you will go down this instant." He got through at last, without doing or saying any thing ridiculous, in which respect he was luckier than another stalwart bridegroom of my acquaintance, who was so dazed and overcome that he held out one of his own fingers for the ring when the minister said: "With this ring I thee wed." Another bridegroom I know lost his head to such a degree that, when it came time for him to say "I, Horace, take thee, Annie, to be my lawful wedded wife," he said, in an unnaturally loud tone; "I, Annie, take thee, Horace, to be my lawful wedded wife," and when the time came for him to introduce his bride to some of his friends who had not yet seen her, he did it by saying, awkwardly, "Ah, er—Miss Carter, this is my wife, Miss Barton," calling her by her maiden name. Few men say "my wife" easily and naturally the first time they use the words in public. A funny case was that of a badly rattled bridegroom who stared blankly at the minister until asked if he took "this woman to be his lawful wedded wife," when he started and said, in the blandest manner: "Beg pardon, were you speaking to me?" A village preacher said that he once married a rural couple at the home of the bride's parents in the presence of a large company of invited guests. The bridegroom was a big, bony, red faced young fellow, who looked as though he could fell an ox with his fist; but he shivered and turned pale at the beginning of the ceremony, and at its close fell down in a dead faint, to the manifest annoyance of his bride, who had been as cool as a cucumber.

